# Inaccuracy of Enzyme-Linked Immunosorbent Assay Using Soluble and Recombinant Antigens to Detect Asymptomatic Infection by *Leishmania infantum*

**DOI:** 10.1371/journal.pntd.0000536

**Published:** 2009-10-20

**Authors:** Elizabeth Castro Moreno, Andréa Vieira Gonçalves, Anderson Vieira Chaves, Maria Norma Melo, José Roberto Lambertucci, Antero Silva Ribeiro Andrade, Deborah Negrão-Corrêa, Carlos Mauricio de Figueiredo Antunes, Mariângela Carneiro

**Affiliations:** 1 Departamento de Parasitologia, Instituto de Ciências Biológicas, Universidade Federal de Minas Gerais, Programa de Pós-Graduação em Parasitologia, Belo Horizonte, Minas Gerais, Brasil; 2 Fundação Nacional de Saúde, Belo Horizonte, Minas Gerais, Brasil; 3 Faculdade de Medicina ,Universidade Federal de Minas Gerais, Programa de Pós-Graduação em Ciências da Saúde: Infectologia e Medicina Tropical, Belo Horizonte, Minas Gerais, Brasil; 4 Centro de Desenvolvimento da Tecnologia Nuclear, Comissão Nacional de Energia Nuclear, Belo Horizonte, Minas Gerais, Brasil; Institute of Tropical Medicine, Belgium

## Abstract

**Background:**

One of the most important drawbacks in visceral leishmaniasis (VL) population studies is the difficulty of diagnosing asymptomatic carriers. The aim of this study, conducted in an urban area in the Southeast of Brazil, was to evaluate the performance of serology to identify asymptomatic VL infection in participants selected from a cohort with a two-year follow-up period.

**Methodology:**

Blood samples were collected in 2001 from 136 cohort participants (97 positive and 39 negatives, PCR/hybridization carried out in 1999). They were clinically evaluated and none had progressed to disease from their asymptomatic state. As controls, blood samples from 22 control individuals and 8 patients with kala-azar were collected. Two molecular biology techniques (reference tests) were performed: PCR with *Leishmania*-generic primer followed by hybridization using *L. infantum* probe, and PCR with specific primer to *L. donovani* complex. Plasma samples were tested by ELISA using three different antigens: *L. infantum* and *L. amazonensis* crude antigens, and rK39 recombinant protein. Accuracy of the serological tests was evaluated using sensitivity, specificity, likelihood ratio and ROC curve.

**Findings:**

The presence of *Leishmania* was confirmed, by molecular techniques, in all kala-azar patients and in 117 (86%) of the 136 cohort participants. Kala-azar patients showed high reactivity in ELISAs, whereas asymptomatic individuals presented low reactivity against the antigens tested. When compared to molecular techniques, the *L. amazonensis* and *L. infantum* antigens showed higher sensitivity (49.6% and 41.0%, respectively) than rK39 (26.5%); however, the specificity of rK39 was higher (73.7%) than *L. amazonensis* (52.6%) and *L. infantum* antigens (36.8%). Moreover, there was low agreement among the different antigens used (kappa<0.10).

**Conclusions:**

Serological tests were inaccurate for diagnosing asymptomatic infections compared to molecular methods; this could lead to misclassification bias in population studies. Therefore, studies which have used serological assays to estimate prevalence, to evaluate intervention programs or to identify risk factors for *Leishmania* infection, may have had their results compromised.

## Author Summary

Visceral leishmaniasis or kala-azar in Brazil, caused by a parasite protozoon, is transmitted to humans by sandflies and has wild and domestic dogs as reservoirs. The disease can become chronic, characterized by fever, weight loss, and enlargement of the liver and spleen. However, the infection in humans can be asymptomatic, and the importance of this phase is unknown. The low levels of immune response and the small number of circulating parasites in the blood are the main problems in identifying the asymptomatic phase. In this study we evaluated the performance of the serology tests compared with molecular tests to identify asymptomatic infection in individuals identified in an urban area of Brazil. Also included were non-infected individuals (healthy controls) and patients (diseased controls). Blood was collected and examined by different serological methods and compared with molecular techniques. The presence of the parasite was confirmed by all molecular techniques and should be the method used to identify true infection. The serology methods showed low sensitivity in detecting the infection. We concluded that the serologic methods are inaccurate in identifying asymptomatic infection when compared to molecular techniques and could underestimate the infection in population studies.

## Introduction

The global number of new human cases of visceral leishmaniasis (VL) or kala-azar is estimated to be about 500,000 per year; Bangladesh, Brazil, India, Nepal, Ethiopia and Sudan account for approximately 90% of the estimated global disease prevalence [1]. In Brazil, VL is caused by *Leishmania (Leishmania) infantum ( = L.chagasi)*, a zoonotic infection transmitted mainly by *Lutzomyia (Lutzomya) longipalpis*, having as reservoirs wild and domestic canids. Initially, it was characterized as an endemic infection from rural areas, occurring primarily at Northeastern States of Brazil. During the last decade, an increasing number of clinical VL is being reported from Brazilian large cities, such as the metropolitan region of Belo Horizonte, Minas Gerais State capital. VL incidence rates in this State, located in the Southeast region of Brazil, were 2.6 and 2.1/100.000 population in 2005 and 2006, respectively [2].

Visceral leishmaniasis is a chronic and progressive systemic disease characterized by fever, weight loss and hepatosplenomegaly; it may lead to death if left untreated. To control the disease, early diagnosis and treatment are essential; however, due to the lack of specificity of VL clinical symptoms, the infection should be confirmed by parasite finding or antibody-detection tests. VL induces a strong B cell activation resulting in hypergammaglobulinemia [3]. Antibody-detection tests, such as direct agglutination test (DAT), immunofluorescence antibody test (IFAT) and enzyme-linked immuno-fluorescent assay (ELISA) have shown high diagnostic accuracy in confirming suspected cases of clinical VL.

Most soluble antigens used for ELISA assays are derived from promastigotes of different species of *Leishmania* cultivated *in vitro*, especially *L.amazonensis*, specie that can be easily grown under *in vitro* conditions [3]. The rK39 recombinant protein that is conserved within the *L.donovani* complex has also been used in ELISA tests with high sensitivity and specificity [4].

*L.infantum* infection in humans does not always result in clinical illness. A large proportion of asymptomatic VL has been reported from areas of parasite transmission [5]–[9]. In endemic areas of Brazil, asymptomatic infections are far more numerous than clinical VL; however the relevance of asymptomatic infection in parasite transmission and disease outcome is mostly unknown. Based on serology, the prevalence of asymptomatic infection in population studies ranged from 2.4 to 14% [6],[10],[11]. One of the most important drawbacks in these studies is the difficulty of diagnosing asymptomatic VL patients, probably due to the fact that they present low levels of antibodies and minute amounts of circulating parasites, compared to symptomatic patients [12],[13]. This would explain the conflicting results from studies using leshmanin skin test and serology in diagnosing asymptomatic VL patients [10],[14],[15]. Molecular biology techniques, such as polymerase chain reaction (PCR) alone or in combination with hybridization have been used to confirm the diagnosis of VL in suspect cases. These techniques present high sensitivity (ranged from 75.0 to 98.0%) and specificity (ranged of 97 to 100%) to identify the infection in individuals with low parasite burden, low antibody reactivity and absence of symptoms [13], [16]–[18].

The aim of the present investigation was to evaluate the performance of ELISA assays, (using *L.infantum* and *L.amazonensis* promatigotes crude antigens and rK39 recombinant protein), compared to PCR and hybridization (reference tests) to diagnose asymptomatic VL infection in participants from a cohort study with a two-year follow-up period.

## Material and Methods

### Study design and population

The study was approved by the Ethical Review Board of the Universidade Federal de Minas Gerais (No. 081/98). All participants or their legal guardians were required to sign the Informed Consent Form before data collection.

The participants (136) were selected from a population-based cohort of individuals, being followed since 1999, living in an area with active *L.infantum* transmission (urban area of General Carneiro, Sabará City, Metropolitan Region of Belo Horizonte, capital of Minas Gerais State, Brazil). At baseline, 97 and 39 individuals, according to PCR/hybridization results, were classified as positive or negative for *L.infantum* infection, respectively; all positive participants were asymptomatic [6]. They were re-evaluated in 2001 (present investigation); during this period, none of the infected participants had progressed to clinical disease. Blood samples from 22 non-infected individuals and from 8 patients with clinical VL, all of them residents of the metropolitan region of Belo Horizonte, were also assayed for comparison and laboratory techniques quality control.

### Clinical exams

The participants were clinically examined for signs and symptoms of VL; anthropometric measures were also recorded. Results were registered in a standardized protocol.

### Sample collection

Blood samples were collected in vacuum tubes containing EDTA. After centrifugation, plasma was aliquoted and kept at −20°C until use in ELISA tests. Peripheral blood mononuclear cells (PBMC) were isolated in Ficoll-Paque gradient and also kept at −20°C until be used for DNA extraction.

### Antigens

Each plasma sample was tested against three different *Leshmania*-antigen preparations: crude extract from *L.infantum*, crude extract form *L.amazonensis* and rK39 recombinant antigen. For antigen preparation, *L.infantum* (MHOM/BR/1972/BH46) or *L.amazonensis* (MHOM/BR/1960/BH6) promastigotes were grown to stationary phase in LIT (Liver Infusion Tryptose) medium. The crude antigens were prepared as previously described [19]. The rK39 antigen was prepared according to procedures described by Burns et al. [20] and provided by Heska Corporation, USA.

### ELISA-assay

*Leshmania*-reactive IgG1 antibody, the predominant IgG isotype in VL infection [21] was tested by ELISA in the plasma samples. The assays were performed using the methodology described before [19]. Reactive antibody was detected with horseradish peroxidase-conjugated anti-human IgG1, (Sigma), diluted 1∶1000 in PBS. The absorbance was measured in a microplate reader (Labsystems Multiskan RC) at 492 nm. The cut-off values were established for each antigen tested, and calculated based on the mean of absorbance value plus 2 standard deviations from 22 plasma samples of healthy control group. Therefore, reactive samples had OD superior to 0.085 when tested against *L.amazonensis* crude antigen, 0.032 against *L.infantum* crude antigen and 0.039 against rK39.

### Molecular techniques

The DNA was extracted from PBMC of each participant and stored at 4°C [6]. Two PCR protocols were performed. In the first protocol, a sample of DNA was amplified using a pair of generic primers [5′-(G/C)(G/C)(C/G) CC(A/C) CTA T(A/T) T TAC ACC AAC CCC - 3′ and 5′-GGG GAG GGG CGT TCT GCG AA – 3′] described by Degrave et al. [22] that amplify a fragment of 120 bp of the conserved region of *Leishmania* kDNA minicircle. The PCR reaction was done as specified by Moreno et al. [6]. The reaction products were visualized in 5% polyacrylamide gel electrophoresis stained with silver and a 100-bp DNA ladder (Gibco BRL) was used as marker. These amplified DNA were denatured, applied on nylon membranes (Biodyne A, Gibco BRL) using a bio-dot apparatus (Hybri-dot manifold-BRL) and hybridized using probes composed of cloned minicircles from *L.infantum* radiolabeled with 32P - [α]dCTP, as previously described, to confirm the infection by a *Leishmania* of the donovani complex [22],[23].

In the second protocol, to confirm *L. infantum* infection, a PCR was performed in the same samples of DNA using a pair of primers [5′- ACG AGG TCA GCT CCA CTC C-3′ and 5′-CGT AGA CAC AGG CGT TGC AG-3′] that amplify a fragment of 100 bp and is specific to *L.donovani* complex. The PCR reaction was done according to Piarroux et al. [24].

Appropriated positive controls, consisting of promastigote DNA, and samples with no DNA as negative controls were used in all performed reactions. To confirm DNA integrity, negative DNA samples were also tested for the human ß-globin gene using [5′- CAA CTT CAT CCA CGT TCA CC- 3′ and 5′-ACA CAA CTG TGT TCA CTA GC- 3′] as primers. Procedures to avoid carryover contamination from previously amplified DNA were used routinely.

### Anti-*Trypanosoma cruzi* antibodies

Due to cross-reactivity in serological tests, serum samples were also assayed for *Trypanossoma cruzi* antibodies using indirect hemagglutination (Chagas Kit -FUNED-SES/MG, Brazil) and IFAT (Chagas Kit –BioManguinhos – FIOCRUZ, Brazil).

### Reproducibility

Ten percent of blood samples and DNA samples were randomly selected to be masked re-tested; each duplicate received a different number from the original sample.

### Statistical analysis

Tables of contingency, χ^2^ and Fisher exact test were used to compare the diagnosis tests. Non parametric tests (Kruskal Wallis and Dunn test) were used to compare the quantitative results. The validity of the serological tests was estimated by the sensitivity, specificity and likelihood ratios using the results of molecular techniques (PCR/hybridization) as the reference standard. The ROC curve was used to estimate the performance of each antigen to identify the infection. The agreement between qualitative tests was estimated by the kappa statistic [25].

## Results

Participants mean and median age were 34.0±20.6 and 32 years (IQR 14, 51), respectively; females comprised 54.4% of the sample. The mean time of residence in the region was 18±11.3 years.

All assays (molecular and serological tests) were negative for the 22 healthy controls and positive for the 8 patients with kala-azar.

**Figure 1:**
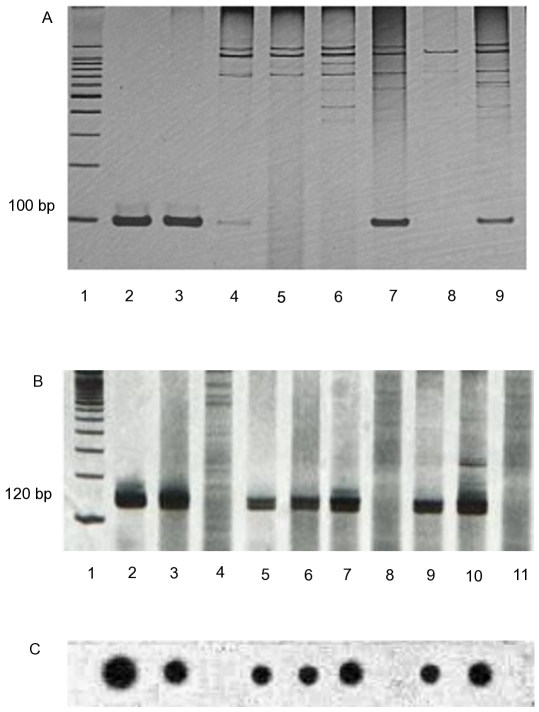
PCR and hybridization products to identify *L. donovani* complex in blood samples. A: PCR products obtained with primers specific for the *L. donovani* complex (Piarroux, 1993); molecular size markers (lane 1), positive control (lane 2-kDNA extracted from cultured *L.infantum*), VL patient (lane 3), asymptomatic (lanes 4;7;9), health individuals (lane 5–6); negative control (lane 8- no DNA). B: PCR products obtained with primers for genus *Leishmania* (*Degrave et al 1994*); molecular size markers (lane 1), VL patient (lane 2), positive control (lane 3-kDNA extracted from cultured *L. chagasi*); healthy individual (lane 4), asymptomatic (lanes 5–7, 9–10), negative control (lanes 8 and 11- no DNA). C: Dot-blots obtained using specific probe for the *L. donovani* complex, hybridized with PCR products.

PCR results for 136 participants showed 117 positive (*Leishmania* primers; amplified product hybridized with radiolabed probe specific for *L.donovani* complex) and 19 negative. Similar results were obtained with the second PCR protocol (specific primers for the *L.donovani* complex), showing 100% agreement. An illustrative example of the PCR products obtained from the amplification of DNA extracted from PBMC of negative-control, VL symptomatic patients and some participants of the cohort study, using each PCR procedure, can be seen in Figure 1.

It should be emphasized that (1) among the 117 PCR/hybridization positive participants, 80 were positive at baseline and 37 became positive during follow-up; (2) among the 19 PCR/hybridization negative, 17 were positive at baseline and became negative, and 2 remained negative during follow-up; (3) none developed clinical VL during follow-up (see Table 1).

**Table 1:** Positive tests for *Leishmania infantum* infection among the 136 cohort participants at baseline and 24 months of follow-up. General Carneiro, Minas Gerais.

Diagnostic Test	Positive at Baseline evaluation	Follow-up		
		Remained positive (%)(1)	Became positive (%)(2)	Total of positive (%)
PCR/hybridization	97/136 (71.3)	80/97 (82.5)	37/39 (94.8)	117/136 (86.0)
*L. amazonensis*-ELISA	82/136 (60.2)	49/82 (59.8)	18/54 (33.3)	67/136 (49.3)
*L. infantum*-ELISA	53/136 (40.0)	32/53 (60.4)	28/83 (33.7)	60/136 (44.1)
rK39-ELISA	39/136(28.7)	15 /39(38.5)	21/97 (21.6)	36/136 (26.5)

	Negative at Baseline evaluation	Remained negative (%)(2)	Became negative (%)(1)	Total of negative (%)
PCR/hybridization	39/136 (28.7)	2/39 (5.1)	17/97 (17.5)	19/136 (14.0)
*L. amazonensis*-ELISA	54/136 (39.8)	36/54 (66.7)	33/82 (40.2)	69/136 (50.7)
*L. infantum*-ELISA	83/136 (60.0)	55/83 (66.3)	21/53 (39.2)	76/136 (55.9)
rK39-ELISA	97/136 (71.3)	76/97 (78.4)	24/39 (61.5)	100/136 (73.5)

(1) among those positive at baseline.

(2) among those negative at baseline.

**Figure 2:**
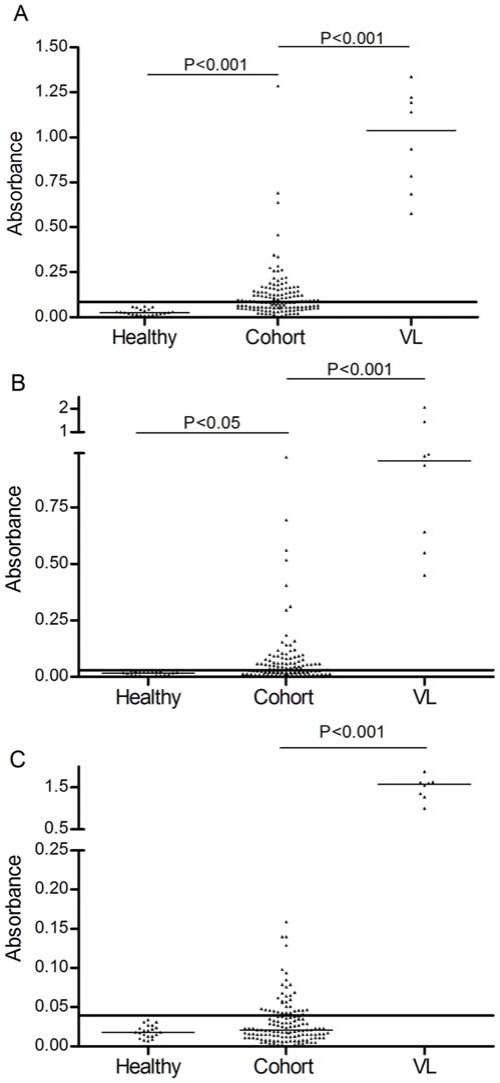
Comparison of absorbances between cohort's participants, positive and negative controls, using three different antigens. A: *L. amazonensis.* B: *L.infantum.* C, rK39. Healthy = 22 negative controls; Cohort = 136 inhabitant of endemic area; VL = 8 positive controls (patients with visceral leishmaniasis). Differences statistically significant were sign (p<0.05, p<0.001, Kruskall Wallis and Dunn's test).

Regarding serological assays, higher median absorbance values were observed comparing the 136 cohort participants with healthy controls (Kruskall Wallis, p<0.001) using both *L.amazonensis* and *L.infantum* crude antigens; this difference was not observed when rK39 antigen was used. Moreover, median absorbance values for all antigens were lower among cohort participants compared to diseased controls (Kruskall Wallis, p<0.001, Figure 2). The same results were observed when the comparisons were done without the 19 PCR-negative cohort participants (data no shown).

In contrast with the molecular diagnosis, serological evaluation of the 136 participants showed that only 67 (49.3%) plasma samples were reactive for *L.amazonensis* antigen, 60 (44.1%) for *L.infantum* and 36 (26.5%) for rK39. The evaluation of serological results at the follow-up is shown in Table 1: (1) among the positive participants in the first evaluation, 59.8% remained positive in *L.amazonensis*-ELISA, 60.4% in *L.infantum*-ELISA and 38.5% in rk39-ELISA; (2) among the negative participants in the first evaluation, 33.3% became positive in *L.amazonensis*-ELISA, 33.7% in *L.infantum*-ELISA and 21.6% in rk39-ELISA.

The agreement between the serological tests is shown in Table 2; poor kappa (<0.10) was observed for all comparisons. No significant differences regarding tests agreement were detected when the cut-off value for each antigen was modified from two to three standard deviations (data not shown).

**Table 2:** Agreement between crude (*L.amazonensis*, *L.infantum*) and recombinant antigens (rK39) in sera from cohort participants, General Carneiro, Minas Gerais.

Comparisons	Agreement		Total Agreement (%)	Kappa (95% CI)
	Positive in both (%)	Negative in both (%)		
*L amazonensis* vs. *L. infantum*	33 (24.3)	42 (30.9)	75 (55.1)	0.10 (−0.07–0.269)
*L amazonensis* vs. rK39	21 (15.4)	54 (39.7)	75 (55.1)	0.10 (−0.05–0.246)
*L. infantum* vs. rK39	15 (11.0)	55 (40.4)	70 (51.5)	−0.03 (−0.183–0.128)

Denominators of the estimated proportions were 136 participants.

The evaluation of tests reproducibility (duplicates) showed an excellent agreement for ELISA-*L.infantum* (kappa 0.88, 95% CI 0.39–1.4), good for ELISA-*L.amazonensis* (kappa 0.69, 95% CI 0.24–1.13) and good for rK39 (kappa 0.77 95% CI 0.53–1.00).

Table 3 shows the comparison of serological and molecular results. Sensitivity and specificity were estimated using the molecular methods as reference. The crude antigens showed higher sensitivity (49.6% and 41% for *L.amazonensis* and *L.infantum* respectively) than rK39 (26.5%); rK39 was more specific (73.7%) than *L.amazonensis* (52.6%) and *L.infantum* (36.8%). The likelihood ratio for each antigen showed a lower power to determine the occurrence of the infection. The combined sensitivity and specificity [25] for serological assays were 80.4% and 14.3%, respectively.

**Table 3:** Validity of serological tests in comparison with molecular test to identify *L. infantum* infection of 136 participants of a cohort study, General Carneiro, Minas Gerais.

Serological Test	TP	FP	FN	TN	Sensitivity (%) (95% CI)	Specificity (%) (95% CI)	LR+ (95% CI)	LR− (95% CI)	Kappa
*L. amazonensis*	58	9	59	10	49.6 (40.3–58.9)	52.6 (29.5–74.8)	1.1 (0.8–1.4)	1.0 (0.8–1.2)	0.01
*L. infantum*	48	12	69	7	41.0 (32.1–50.5)	36.8 (17.2–61.4)	0.7 (0.5–0.8)	1.6 (1.0–2.7)	−0.10
rK39	31	5	86	14	26.5 (19.0–35.6)	73.7 (48.6–89.9)	1.0 (0.6–1.8)	1.0 (0.9–1.7)	−0.00

Molecular techniques were used as reference test. Criteria: Positive results = positive in PCR with generic primer and specific primer for *L.donovani* complex and positive in hybridization. Negative results = negative in all molecular techniques.

TP = True Positive; FP = False positive; FN = False Negative; TN = True Negative; LR+ = Likelihood test positive; LR− = Likelihood test negative.

The serological tests ROC curves were uninformative. The areas under the curve for each antigen were: Elisa-*L.infantum* = 0.36 (95% CI 0.22–0.50); Elisa-*L.amazonensis* = 0.57 (95% CI 0.42–0.72); Elisa-rk39 = 0.53 (95% CI 0.39–0.68).

The simultaneous response to the antigens used can be seen on Table 4. Among the 117 individuals who tested positive in the molecular biology techniques, only 6 (5.1%) responded simultaneously to the three antigens, whereas 31 (16.5%) did not test positive in any of them; 86 (73.5%) had at least one positive serology. On the other hand, 15/19 (78.9%) molecular biology negative participants tested positive for at least one of the three antigens.

**Table 4:** Comparison between serological results according to the reactivity to different antigens with molecular techniques results, General Carneiro, Minas Gerais.

Serological Results	Molecular Techniques		Total (%)
	Positive(4) (n = 117)	Negative(5) (n = 19)	
Positive test with three antigens(1)	6	1	7(5.1)
Positive tests with two antigens(2)	39	9	48(35.3)
Positive test with one antigen(3)	41	5	46(33.8)
Negative tests with three antigens	31	4	35(25.7)
Total	117	19	136

(1) *L. amazonensis*, *L. infantum* and rK39.

(2) *L. amazonensis* and *L. infantum*, *L. amazonensis* and rK39, *L. infantum* and rK39.

(3) *L. amazonensis* or *L. infantum* or rK39.

(4) Positive = Infected (positive PCR with two primers and confirmed by hybridization).

(5) Negative = No infected (negative in molecular techniques).

Six participants were identified as *T.cruzi* infected (IFAT and Indirect Hemagglutination). They were also positive in molecular techniques (*L.donovani* complex probe), suggesting co-infection.

The demographic and clinical characteristics of the infected and reactive individuals to at least one serologic test (n = 86) were compared to those of non-reactive individuals (n = 31). Among the characteristics evaluated (gender, age, time in which the person has been living in the study area, nutritional status, hemogram results and clinical findings) only age was different among the groups. The infected and seroreactive individuals were older (median = 42 years, IQR = 18, 52) than the nonreactive infected individuals (median = 17 years, IQR = 12, 29).

## Discussion

In this study, a perfect agreement was observed comparing PCR conducted with two different primers and confirmed with specific probe for donovani complex, indicating a high accuracy in identifying *L.infantum* infection. Therefore, molecular techniques were used as reference tests in the evaluation of this infection among cohort participants. During follow-up, no participants developed symptomatic VL, although most of them had remained positive in molecular assays.

On the other hand, the serologic methods showed to be inadequate and inaccurate in identifying *L.infantum* asymptomatic infection. The crude antigens and the recombinant antigen used showed low validity, i.e. none was able to identify all infected individuals. In addition, high disagreement was found when they were compared, although good and excellent agreements were observed when their reproducibility was tested.

Serological assays showed significant lower absorbancy levels in diagnosing asymptomatic individuals compared to kala-azar patients. These results indicate the occurrence of low levels of antibodies in the blood of these individuals, which was already suggested by different authors [12],[13]. Regardless the fact that most cohort participants have maintained their positivity (molecular techniques) the serological tests showed low reactivity reinforcing the results from the cohort baseline [6].

Our data suggest that homologous and heterologous antigens have different accuracy in diagnosing *L.infantum* infection. Plasma which detect homologous antigen may not detect heterologous antigen or vice-versa. The rK39 recombinant antigen presented the lowest sensitivity and the highest specificity in identifying asymptomatic individuals, which was already recognized in different studies [4],[26].

Only 5.1% (6/117) of the infected individuals were simultaneously positive in all antigens. However, using all possible combinations, it is possible to identify 73.5% (86/117) of the infected individuals. Therefore, the use of more than one serological diagnostic method increases the probability to recognize asymptomatic individuals [8],[27]. But it should be considered that, due the low specificity of serological methods, false-positive results could be included. In this study, the percentage of false-positive in the serology was quite high (78.9% = 15/19).

It is known that tests performed in parallel increase the sensitivity and decrease the specificity of the diagnosis. Our results of combined sensitivity and specificity (80.4% and 14.3%, respectively) confirmed the tests low accuracy in identifying asymptomatic carriers.

Serological tests poor concordance may be explained by the low level of antibody production, due to the small number of parasites observed in asymptomatic carriers when compared to clinical patients [12],[13] and the performance of crude and recombinant antigens in identifying the infection. Crude antigens derived from promastigotes cultivated in vitro consist of a repertoire of somatic antigens and several surface components that vary according to parasite species used as antigens [3].

Total soluble antigens or recombinant antigens, traditionally used in ELISA tests, are adequate to confirm the diagnosis in a suspect VL case. In addition, it has been recognized that accuracy is not a fixed test property; it can vary with the clinical settings and with the spectrum of disease (spectrum bias). Therefore, in field conditions and in individuals with low level of humoral immune response, test performance can be affected [8],[15],[27]. Our limitation in evaluating the accuracy of serological tests was the small number of individuals not infected in the study population; the measurements had wide confidence intervals.

Age was the only risk factor associated to the different antigens reactivity: infected individuals positive in at least one antigen were older (median = 42 years) than those non-reactive (median = 17 years). The meaning of this finding deserves further investigation.

Diagnostic tests results can be questioned due the possibility of cross reactivity with other parasite infections in the studied population. Low incidence rate of cutaneous leishmaniasis, caused by *Leishmania braziliensis*, has been reported from Sabara City. However, in our study, during the anamnesis and clinical examination, cohort participants did not present any clinical symptoms or report a history of cutaneous disease. Moreover, molecular techniques used in our experimental protocol are specific for *L.donovani* complex (*L.infantum* in the New World) diagnosis, therefore excluding *L.braziliensis* infection.

As pointed out in our results, six participants were co-infected with *L.infantum* and *T.cruzi*. Although cross-reactivity among *Leishmania* and *T.cruzi* antigens has been reported, it is important to note that molecular techniques used herein are specific for *L. donovani* complex; in addition the studied population showed low reactivity in ELISA tests. The proportion of *T.cruzi-L.infantum* co-infected participants did not interfere in the analysis of asymptomatic infection.

This study has the advantage of using molecular techniques to correctly identify infected individuals; most studies on asymptomatic used only one serological test or skin tests. Positive molecular methods suggest the presence of living parasites. Recent studies by microscopy and real-time PCR demonstrated that *Leishmania* DNA detected by PCR is derived from intact parasites; DNA is rapidly degraded following amastigote death. Positive result is correlated to the presence of living parasites, while a negative result is obtained after parasitological cure [28].

Notwithstanding the high agreement observed between molecular techniques and the persistence of its positivity in most cohort participants, important questions remain to be answered: (1) what was the meaning of a positive PCR in individuals that did not progressed to symptomatic VL during the follow-up? (2) why some participants, living in a transmission area, changed their status from positive to negative? To answer these questions, a longer follow-up period may be necessary in order to understand the role of a persistent positive PCR in the disease process. It should also be considered that participants live in transmission areas, being exposed to re-infection.

Why it is important to use an accurate test in identifying an asymptomatic infection? Firstly, to obtain reliable estimate of infection occurrence in endemic and in recent transmission areas. This information will be useful to prioritize and to evaluate control measures. Currently, there is no evidence that infected or asymptomatic individuals can act as *L.infantum* host in urban areas; the probability of a phlebotomine being infected when feeding in an asymptomatic individual seems to be very low [29]. However, it is important to consider the number of asymptomatic individuals in urban areas and the environmental conditions which may favor man-vector transmission [8],[9],[27],[30]. Secondly, infected individuals may become immunocompromised, altering their course of infection and parasitaemia [31].

In conclusion, molecular techniques are better suited for evaluation studies, as they can minimize misclassification bias in population based investigations. Serological tests can not be reliably used as a tool to identify asymptomatic individuals, especially when only one antigen is used. In the impossibility of carrying out molecular tests, one should weigh the consequence of using serological techniques to identify asymptomatic carriers in population studies: probably, around 20 to 30% of infected individuals will be missed. On the other hand, to carry out studies using PCR with a large number of samples under field conditions is difficult and expensive; the feasibility of this approach needs to be better appraised. Further studies to develop and to validate new diagnostic methods for asymptomatic *L.infantum* infection are urgently needed; these new tools are essential in population based investigations: they should allow the valid and reliable identification of all infected individuals.
